# Crosstalk among Calcium ATPases: PMCA, SERCA and SPCA in Mental Diseases

**DOI:** 10.3390/ijms22062785

**Published:** 2021-03-10

**Authors:** Tomasz Boczek, Marta Sobolczyk, Joanna Mackiewicz, Malwina Lisek, Bozena Ferenc, Feng Guo, Ludmila Zylinska

**Affiliations:** 1 Department of Molecular Neurochemistry, Medical University of Lodz, 92215 Lodz, Poland; tomasz.boczek@umed.lodz.pl (T.B.); marta.sobolczyk@stud.umed.lodz.pl (M.S.); joanna.mackiewicz1@stud.umed.lodz.pl (J.M.); malwina.lisek@umed.lodz.pl (M.L.); bozena.ferenc@umed.lodz.pl (B.F.); 2 Department of Pharmaceutical Toxicology, China Medical University, Shenyang 110122, China; blueforest611@hotmail.com

**Keywords:** calmodulin, calcium, plasma membrane Ca^2+^-ATPase, sarco/endoplasmic Ca^2+^-ATPase, secretory pathway Ca^2+^-ATPase, mental diseases

## Abstract

Calcium in mammalian neurons is essential for developmental processes, neurotransmitter release, apoptosis, and signal transduction. Incorrectly processed Ca^2+^ signal is well-known to trigger a cascade of events leading to altered response to variety of stimuli and persistent accumulation of pathological changes at the molecular level. To counterbalance potentially detrimental consequences of Ca^2+^, neurons are equipped with sophisticated mechanisms that function to keep its concentration in a tightly regulated range. Calcium pumps belonging to the P-type family of ATPases: plasma membrane Ca^2+^-ATPase (PMCA), sarco/endoplasmic Ca^2+^-ATPase (SERCA) and secretory pathway Ca^2+^-ATPase (SPCA) are considered efficient line of defense against abnormal Ca^2+^ rises. However, their role is not limited only to Ca^2+^ transport, as they present tissue-specific functionality and unique sensitive to the regulation by the main calcium signal decoding protein—calmodulin (CaM). Based on the available literature, in this review we analyze the contribution of these three types of Ca^2+^-ATPases to neuropathology, with a special emphasis on mental diseases.

## 1. Introduction

**Figure 1:**
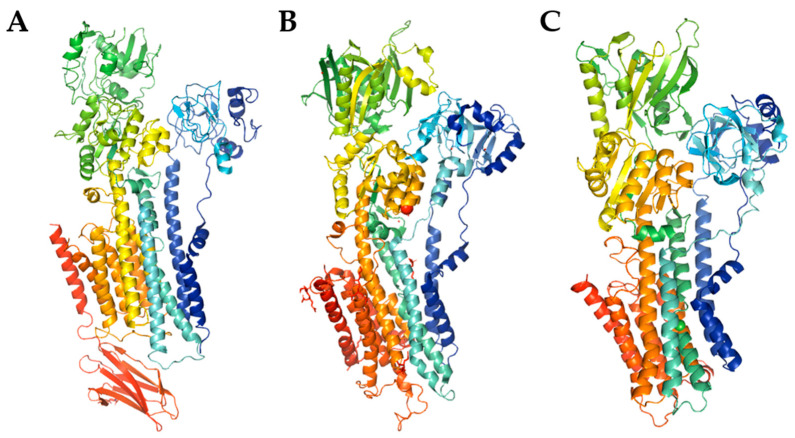
The model of PMCA ((**A**), PDB entry code 6A69) and SERCA ((**B**), PDB entry code 3W5C) structures, and SPCA structure prediction using SWISS-MODEL (**C**). The cartoon models were generated with PyMOL.

Ca^2+^-ATPases are key components of Ca^2+^ extrusion machinery and thus are pivotal for preservation of neuronal function. Among three main calcium pumps, the plasma membrane Ca^2+^-ATPase (PMCA) and sarco/endoplasmic Ca^2+^-ATPase (SERCA) are known for decades while the secretory pathway Ca^2+^-ATPase has been discovered in 2000s by two independent laboratories that described novel mutations leading to Hailey-Hailey disease [1,2,3]. All pumps have high affinity for Ca^2+^ and function to restore cytosolic Ca^2+^ concentration [Ca^2+^]_c_ to the resting, nanomolar level following neuronal stimulations. They belong to the superfamily of mammalian P-type ATPases and are characterized by formation of a phosphorylated enzyme intermediate during catalytic cycle [1]. However, they have a low (~15%) degree of sequency identity [1], and differ in several other key features including tissue distribution, regulatory mechanisms, and contribution to neuronal Ca^2+^ homeostasis. Each pump is encoded by multiple genes giving rise to a number of isoforms and further splice variants, which often possess distinguishable kinetic parameters and are dedicated to unique and highly regulated neural processes [4]. Naturally, the pumps share essential basic properties such as membrane topology, catalytic mechanism and probably the general features of 3D structure [5,6], although the structure of SPCA pump has not been solved yet (Figure 1). The rapid expansion of the knowledge on pumps peculiar role, which run parallel to the advances in neuronal Ca^2+^ signaling, led to the identification of several diseases associated either directly or indirectly with Ca^2+^ pumps malfunction. Most of these defects have genetic background and the number of studies have been aimed to characterize their severity, effect on neuronal Ca^2+^ homeostasis and signaling as well as neuronal survival. Besides known neuropathologies, defects in Ca^2+^ pumps and alterations in the mechanisms regulating their activity may also produce subtle, tissue-specific disturbances that are not clinically manifested, yet they may affect neuronal machinery controlling and processing Ca^2+^ signal. In this review, we focus on the contribution of PMCA, SERCA and SPCA to mental diseases and give a special emphasis to altered regulation by calmodulin (CaM) that often accompanies pump defects.

## 2. Calmodulin—Ubiquitous Ca^2+^ Sensor in Neurons

Most commonly, detection and transduction of Ca^2+^ signals in neurons are orchestrated by ubiquitous messenger called calmodulin. CaM is known as a relatively small (149aa; 16.7 kDa) and highly conserved calcium-binding sensor synthesized in all eukaryotic cells. It is particularity involved in synaptic signaling processes, neurotransmitter release and neuroplasticity by modulation (called “calmodulation”) of a large array of binding partners such as enzymes (e.g., adenylate cyclase, calcineurin, cyclic nucleotide phosphodiesterase, nitric oxide synthase, and certain kinases), transcription factors (e.g., CREB, NeuroD2, NFAT and MEF2) as well as various ion channels and transporters [7,8,9,10].

In human, CaM is encoded by three independent genes CALM1, CALM2, CALM3 located on chromosomes 14q32.11; 2p21; and 19q13.32, respectively, which are collectively transcribed into at least eight mRNAs using different alternative polyadenylation signals (reviewed in [7,11]). Next, the resulting protein is susceptible to undergo various post-translational modifications, mainly phosphorylation on tyrosine (Thr26, Thr29, Thr44, Thr79, Tyr99, Thr117, and Tyr138) and serine (Ser81, and Ser101) sites [12]; acetylation of the N-terminal alanine [13]; trimethylation of the Lys115 [14]; and proteolytic cleavage at the C-terminal domain [15], all collectively regulating CaM biological activity. The crystal structure of mature CaM contains two independently folded lobes (N-lobe and C-lobe) connected by a flexible central α-helical linker, that differ by calcium affinity and kinetics of calcium dissociation. Each of these globular clusters can bind up to two free Ca^2+^ ions via a pair of helix-loop-helix motives (EF-hands) in a cooperative manner (K_d_ = 5 · 10^−7^ M to 5 · 10^−6^ M) [16,17,18]. Because of subtle structural differences between these lobes resulting from evolutionary processes [19], EF hands in the C-lobe exhibit a three- to five times higher affinity for Ca^2+^. However, they possess slower rate of ion binding than the regions of EF hands located in the N-lobe, establishing the broad range of CaM sensitivity to the changes in calcium concentrations in the intracellular space [20]. CaM is susceptible to dramatic structural rearrangements via partially exposed hydrophobic patch on the C-terminal domain which may interact with CaM-binding proteins (CaMBPs) in a Ca^2+^ -free (apo-CaM) state or in partially calcium-saturated forms (two Ca^2+^ ions bound to the C-terminus) [16]. Up to date, over three hundred different calmodulin targets with specific binding sites and unique affinities for CaM, many of which located in the central nervous system (CNS) neurons [21], have been validated and extensively characterized [22]. The analysis of over 80 CaM complexes compiled in the Protein Data Bank (PDB) has revealed that CaM binding sites not always contain defined consensus sequence but rather share some common biochemical and biophysical properties such as high helix-forming propensities, positively charged binding region and the presence of hydrophobic anchor residues [8,22]. Thus, the classification of several CaM-binding motifs is determined by the spacing between these anchor residues as was extensively discussed by Mruk and colleagues [23]. As observed from sequence analysis of several CaMBPs, their IQ motif ([FILV]Qxxx[RK]Gxxx[RK]xx[FILVWY]) with highly conserved amino acid residues at positions 1, 2, 5, 6, 11, and 14 or IQ-like ([FILV]Qxxx[RK]Gxxxxxxxx) motif may also bind CaM in the presence or absence of Ca^2+^ [23,24].

Considering the diversity of CaM interactions and its abundance in the brain (up to 100 μM range) [25], it seems rational to suspect that disruption of these multifunctional interactions regulating Ca^2+^-dependent intracellular signal transduction cascades may be implicated in the development of numerous neuropsychiatric disorders. Moreover, there is increasing evidence suggesting that pathophysiology of these states is intimately related to the disturbed neuronal calcium homeostasis also mediated by ATP-driven pumps located in the plasma membrane, in the membranes of the endoplasmic reticulum (ER), or Golgi compartments.

## 3. Plasma Membrane Ca^2+^-ATPase (PMCA)—The Only Calcium Pump Directly Regulated by Calmodulin

PMCA is one of the most important and sensitive players in maintaining of low resting Ca^2+^ concentration, and ensuring a fast recovery of [Ca^2+^]_c_ to the basal level following neuronal excitation [26]. The enzyme was first described by Schatzmann in 1960s as ATP-powered mechanism that removes calcium from red blood cells [27], whereas further studies revealed the presence of PMCA in other cells, including neurons [28,29,30] Structurally, PMCA comprises of ten transmembrane segments with N- and C- terminal tails both located on the cytosolic site [31]. Most of the regulatory regions including acidic phospholipids, protein kinase C (PKC), protein kinase A (PKA) and the crucial natural activator—CaM, are located at the C- terminus. The important regulatory role of CaM in stimulating of PMCA is associated with increasing the affinity of the pump for calcium and the maximum rate of calcium extrusion. In the activation process, CaM removes the auto-inhibitory C-terminal domain from the active site and releases the enzyme from auto-inhibition [32]. It is also worth mentioning that PMCA is so far the only known calcium pump directly activated by CaM [26].

In mammals, four isoforms of PMCA (PMCA1-PMCA4), structurally similar to each other, have been found [4] but their expression depends on cell type (Table 1). The PMCA1 and PMCA4 are widely expressed in virtually all animal tissues and both play a house-keeping role.

Expression of PMCA2 and PMCA3 is highly restricted to excitable cells and their high concentration has been detected in the CNS [4]. PMCA2 is especially abundant in cerebellar Purkinje cells and granule cells, but it also localizes to the cerebral cortex and hippocampus [33]. PMCA3, in turn, is present predominantly in cerebellar granule cells and in the choroid plexus [34] what suggests its role in generation and release of cerebrospinal fluid. Additionally, PMCA isoforms are characterized by distinct calmodulin sensitivity (Table 1) and specific kinetic properties. PMCA2 and PMCA3 are referred to as “fast” isoforms due to their high basal activity and high affinity for CaM, whereas PMCA1 and PMCA4 are much slower despite their strong stimulation by CaM [1]. It has been suggested that the cell response to a physiological stimulus depends on significant differences in the kinetic parameters of the individual isoforms. In the brain, distribution of PMCA isoforms clearly alters during development, what may indicate their specific role in embryogenesis and further in postnatal period [35].

In addition to control critical neuronal functions such as synaptic transition and neurotransmitter release, neuronal Ca^2+^ also participates in the regulation of survival and differentiation, processes common to other cell types [36]. Early in vitro study on differentiated pheochromocytoma-derived cells, a model frequently used to mimic the physiology of sympathetic neurons, has shown that PMCA1 knockdown impaired neuritogenesis and axonal elongation [37]. Similar effect was seen when PMCA2 or PMCA3 expression was decreased with isoform-specific antisense oligonucleotides [38,39] suggesting a role of PMCA in neuronal differentiation. Moreover, cells deficient in neuron-specific PMCA isoforms were unable to maintain Ca^2+^ and pH homeostasis which translated into altered activity of main signaling- and energy-generating pathways [40,41,42]. There is also a compelling evidence that PMCA4 is of paramount importance for neuronal survival in the conditions of Ca^2+^ overload as pheochromocytoma viability was preserved or impaired when this isoform was overexpressed or downregulated, respectively [43,44]. The sections below further explore the association and the specific role of the PMCA in neurodegenerative disorders.

### 3.1. PMCA in Neuropathology

#### 3.1.1. PMCA in Aging

Contribution of PMCA to age-related neuropathologies was first suggested by Michaelis and coworkers [45,46,47]. These authors showed for the first time that PMCA activity and abundance in the synaptosomal membranes is reduced with age, similar to the PMCA activation by aged CaM. Zaidi and coworkers further demonstrated that the decline in PMCA activity was progressive with increasing biological age and was associated with lowered maximal velocity (V_max_) with no apparent changes in the affinity for Ca^2+^ [48]. The age-dependent alterations in PMCA are likely to be a consequence of oxidative stress as PMCA was identified to be a target for reactive oxygen/nitrogen species as does for CaM [49]. For instance, exposure of purified pump protein to H_2_O_2_ inhibited both basal and CaM-stimulated activity. However, neither CaM binding to the oxidized protein nor the concentration-dependent CaM effect on PMCA were affected, suggesting that C-terminal CaM binding domain is not primarily targeted by the oxidant. Pretreatment of PMCA with CaM almost completely preserved PMCA activity in the presence of H_2_O_2_ indicating that conformational state upon CaM binding may be more resistant to oxidation [50]. Several oxidative agents have been demonstrated to abolish PMCA sensitivity to CaM in a concentration-dependent manner [51], induce proteolytic degradation in synaptic membranes [52] or promote internalization and subsequent lack of detectable PMCA expression in hippocampal neurons [53].

PMCA activity is also affected by lipid-surrounding environment. It was demonstrated that PMCA and CaM are partitioned to the cholesterol-rich lipid rafts and PMCA activity in these membrane microdomains is higher than in non-raft regions [54]. Moreover, raft-localized PMCA is more sensitive to age-dependent loss of the activity [55]. Depletion of cholesterol drastically inhibited the activity of raft-associated PMCA but did not produce any effect on non-raft PMCA [54]. Therefore, increasing lipid order may be beneficial for protection of PMCA activity in the aged membranes but cannot overcome age-dependent loss of PMCA.

#### 3.1.2. PMCA in Alzheimer’s Disease

Besides brain aging, altered PMCA expression and activity was detected postmortem in the brain cortex of patients affected by Alzheimer’s disease (AD) [56,57]. One of the histological hallmarks of this disease are the presence of a senile plaques of the amyloid β-peptide (Aβ) and accumulation of an abnormal tau protein [58]. Biochemical studies have revealed that Aβ decreased the activity of purified PMCA and the strongest inhibitory effect was seen for PMCA4 [57]. Moreover, cholesterol was shown to abolish the inhibitory effect of Aβ and the level of inhibition was lower in the lipid rafts of synaptosomal membranes than in non-rafts [57]. The Aβ inhibitory effect can be blocked by CaM, and the activity of PMCA lacking C-terminal CaM-binding domain was unaffected by Aβ. This antagonistic action of CaM is due to physical association with Aβ [59] or competing for PMCA binding [57]. Hence, CaM can protect PMCA activity by masking Aβ-PMCA interacting sites making them unavailable for Aβ. The accumulated data present a clear link between Ca^2+^, CaM and amyloid plaque formation indicating how the dysregulation in neuronal Ca^2+^ homeostasis and Aβ formation affect each other, and how CaM function in the center of this crosstalk. Interestingly, CaM content in the frontal, temporal, parietal cortex and subjacent white matter in AD was reduced by nearly 66% compared to the normal control brains [60]. The mechanism of progressive decline in PMCA and CaM in AD is unknown. However, the recent study on differentiated pheochromocytoma suggests that PMCA downregulation may be a trigger initiating calcineurin/NFAT-dependent repression of CalmII and CalmIII genes [40]. The altered expression of CaM is thus expected to deepen the physiological decline of PMCA function with increasing age and offer insufficient protection not only against Aβ, but also proteolytic and oxidative pump deactivation.

Growing body of evidence suggests that soluble forms of Aβ and tau cooperate with each other to drive healthy neurons into diseased state and the toxicity of Aβ requires tau [58]. The study of Berrocal and colleagues demonstrated that tau, which hyperphosphorylated form is predominant in neurofibrillary tangles, can directly interact with PMCA and inhibit its activity [56]. In this study, PMCA function was solely affected by this interaction as neither SERCA not SPCA were targeted. In differentiated pheochromocytoma, tau was concentrated in growth cones and interacted with PMCA through its N-terminal projection domain [61]. Overexpression of this amino-terminal fragment, but not full-length protein, suppressed nerve growth factor (NGF)-induced axonal outgrowth [61] establishing tau as a mediator of microtubule-plasma membrane interactions during neuritic development. Tau is also required for Fyn kinase-mediated NMDR receptor activation in the postsynaptic densities [62], which strengthen the interaction between NMDA receptor and postsynaptic density protein 95 (PSD-95) [63]. Considering the recruitment of PMCA via PSD-95 to a close proximity to NMDA receptor-mediated Ca^2+^ entry, a direct physical interaction between PMCA and tau in vivo would complement the contribution of PMCA-tau interaction to Ca^2+^ homeostasis dysregulation in the pathogenesis of AD.

Several early studies demonstrated that association between tau and CaM in vitro is Ca^2+^-dependent and it prevents tau interaction with microtubules [64,65]. Binding to CaM also prevents tau phosphorylation by PKC [66]. Although several CaM-dependent kinases and phosphatases are involved in tau posttranslational modification, for instance Ca^2+^/CaM-dependent protein kinase II (CaMKII), cyclin-dependent kinase 5 or protein phosphatase 2B (PP2B or calcineurin) [67], no recent studies have further expanded the functional significant of direct interaction with CaM and tau in the AD.

#### 3.1.3. PMCA in Parkinson’s Disease

Altered PMCA function may significantly contribute to neuronal Ca^2+^ dyshomeostasis and increase the duration and frequency of intracellular Ca^2+^ spikes which may in turn influence the formation of pathological proteins such as the alpha synuclein in Parkinson’s disease (PD) [68]. This hypothesis is supported by the studies of Brendel and coworkers who demonstrated increased Ca^2+^ level and reduction in PMCA2 expression in primary midbrain neurons and neuroblastoma SH-SY5Y treated with Parkinsonian mimetic 1-methyl-4-phenylpyridinium (MPP) [69]. Interestingly, other Ca^2+^ efflux systems such as SERCA pump or Na^+^/Ca^2+^ exchanger remained largely unaffected. The same authors showed that PMCA2 knockdown with siRNA decreased the survival of mesencephalic neurons, but overexpression significantly increased the resistance of midbrain neurons to MPP toxicity [69]. These data indicate that PMCA2, which possesses the highest affinity for CaM, is particularly vulnerable to the inhibition by MPP. The mechanistic explanation of this phenomenon may lie in the oxidative stress and partial oxidative inactivation of PMCA, as membrane protein-selective antioxidants fully prevented MPP toxicity [70].

PMCA inactivation or even PMCA2 knockdown are known to irreversibly deprive neurons of substantial part of their Ca^2+^ clearing potency leading to dysregulation of Ca^2+^ homeostasis [39,68,71]. It is known that Ca^2+^ is a key controller of synuclein formation and Ca^2+^-dependent binding of CaM to α-synuclein accelerates formation of protein fibrils in vitro [72]. Recent study demonstrated that calcineurin also binds α-synuclein and this interaction is mediated by Ca^2+^/CaM signaling [73]. These and other authors demonstrated that increased calcineurin activity was associated with α-synuclein toxicity [73,74]. The activity of calcineurin is known to be regulated by the interaction with PMCA2 [75] and disruption of this interaction elevates intracellular activity of this phosphatase [76,77]. Although no direct interaction between α-synuclein and PMCA has been established, as does for SERCA pump [78], PMCA by regulating the activity of Ca^2+^-dependent signaling may potentially contribute to PD pathogenesis.

#### 3.1.4. PMCA in Schizophrenia and Bipolar Disorder

Ca^2+^has been placed in the center of dopaminergic hypothesis of schizophrenia, mainly because of its essential role in dopamine receptors D1 and D2-mediated synaptic plasticity and signal transduction [79]. Therefore, it is not surprising that many of calcium signaling proteins, including PMCA and CaM, have been found to be differentially regulated in schizophrenia. In early study, Kluge and Kuhne demonstrated that kinetic properties of CaM-stimulated PMCA were altered in erythrocytes of patients with affective psychoses and hyper- or para-kinetic schizophrenics [80]. Unexpectedly, schizophrenia proteomic studies revealed PMCA4 to be up-regulated in anterior temporal lobe in affected patients [81] what may be seen as compensatory change to counterbalance elevated [Ca^2+^]_c_. On the other hand, proteins such as CaM (CALM1 and CALM2), CaMKII (CAMK2B, CAMK2D, CAMK2G) and CaM-like proteins were found to be downregulated in the brain or secretion fluids [81,82]. These findings limit, but not exclude, the special role of PMCA4 in neuronal signaling in schizophrenia, however the disadvantage of the proteomic studies is a fact they were performed not on isolated cells but on whole tissue. Therefore, it is likely that non-neuronal cells could contribute to revealed changes but because of high sample heterogeneity, these data should be interpreted with caution.

In the subgroup of patients involving mostly those with unipolar maniac and bipolar psychoses, the activation of human erythrocyte PMCA by CaM measured in the presence of sub-optimal concentration of lithium was stronger in all individuals with maniac-depressive episodes [80,83,84]. Interestingly, the activity measured in the conditions of optimal concentration of monovalent ions (Na^+^ and K^+^) was higher in lithium-treated groups than in control and untreated patients suggesting that the CaM-activated PMCA may be differentially regulated in maniac-depressive patients.

The potential involvement of PMCA in schizophrenia is also supported by data derived from pharmacological in vivo models. Recent study on animals challenged with 30 mg/kg ketamine, a drug that is known to mimic a wide spectrum of psychotomimetic and cognitive aberrations observed in schizophrenia in humans [85], demonstrated differential regulation of PMCA expression in functionally distinct brain regions [86]. Moreover, the basal and CaM-stimulated PMCA activities were reduced in the synaptosomal membranes mainly due to a direct interaction of the drug within large catalytic loop and competing with CaM for binding in the C-terminal domain of the pump [87,88]. Similarly, emerging studies also suggest the interaction of ketamine with CaM-dependent enzymes, in particular CaMKII [89,90], but no specific functional studies largely limit the conclusions on CaM and PMCA-mediated Ca^2+^ regulation in schizophrenia.

#### 3.1.5. PMCA in Cerebellar Disorders

Immunohistochemical studies showed that neuron-specific PMCA2 and PMCA3 are more abundant in the cerebellum than PMCA1 and PMCA4. Moreover, they are concentrated in synaptic terminals of Purkinje cells while PMCA1 and PMCA4 localize mostly to granular layer [91]. Purkinje cells integrate the excitatory input to the cerebellar cortex being an essential component for the regulation and coordination of motor movements [92]. It is therefore not surprising that one of the most visible cerebellar dysfunctions during ataxia are uncoordinated movements and inability to maintain body balance. The link between ataxia and PMCA3 became evident when Zanni and coworkers identified a point mutations in ATP2B3 gene (located on the human chromosome X) in a family affected by X-linked congenital cerebellar ataxia [93]. The mutation located in exon 20 of ATP2B3/PMCA3 gene replaces a conserved Gly by Asp in C-terminal CaM-binding domain. The mutated pump demonstrated decreased ability to extrude Ca^2+^ what can be a direct consequence of altered interaction of its C-terminus with CaM. This is supported by mathematical modeling showing reduced ability of CaM to interact with the mutated binding domain what tends to depress the basal PMCA activity and decrease autoinhibitory interaction of CaM-binding domain with the main body of the pump. The possible defective interplay between mutated pump and PMCA-interacting signaling molecules (see Table 2) organized within Ca^2+^ nanodomains could contribute to the phenotype of cerebellar disease. It is even more plausible as more than a dozen X-linked gene defects have been demonstrated to contribute to cerebellar phenotype [94]. Among them, mutation in Ca^2+^/CaM-dependent serine protein kinase (CASK) may be significant [95,96] as CASK interacts with PMCA through PDZ domain located in the C-terminal end of the pump.

Cali and colleagues [97] identified another mutation in ATP2B3 gene in a patient with cerebellar ataxia and global developmental delay. The mutation (R482H) significantly reduced the Ca^2+^ clearing potency of the pump and resulted in the inability to handle intracellular Ca^2+^ transients evoked by cell stimulation. Interestingly, the patient also carried two additional mutations in the LAMA1 gene encoding laminin 1α. It has been well-characterized that mutations or deletions in this gene are associated with cerebellar dysplasia phenotype [98]. Therefore, on the basis of the family pedigree of the patient, it is reasonable to suspect that mutations in PMCA3 along with those in LAMA1 could synergistically contribute to the ataxic symptoms.

Recently, a novel mutation in PMCA3 (G733R) has been identified in a patient carrying a defect in phosphomannomutase 2 (PMM2), indicating a possible link between these mutations in generating ataxia phenotype [99]. PMM2 is an enzyme catalyzing the isomerization of mannose-6-phosphate to mannose1-phosphate and two missense mutations (R123Q and G214S) in PMM2 gene are known to be associated with type I congenital disorder of glycosylation [100,101]. The G733R substitution in the pump disturbed the ability to handle Ca^2+^ rises upon Ca^2+^ release from the ER or influx through the plasma membrane without affecting pump expression or subcellular targeting. The basal activity of autoinhibited pump and constitutive active variant of PMCA3 lacking C-terminal domain was also compromised. The coexistence of both PMCA3 and PMM2 mutations in the patient affected by non-progressive ataxia and muscular hypotonia is of special significance as PMM2 was found to be Ca^2+^-regulated enzyme [102]. Therefore, mutated PMCA3 may give rise to increased Ca^2+^ concentration in microdomains where PMM2 is located, inhibiting it. This, along with the mutations in PMM2, would deepen the decline in PMM2 enzymatic activity.

There is also convincing evidence coming from PMCA2 knockout mice that this isoform, apart from its function in auditory system discussed elsewhere [103,104,105], plays an essential role in cerebellar function. PMCA2^−/−^ mice exhibited severe ataxia that became apparent by 12 days of age and had great difficulty in maintaining body balance [106]. Histological examination showed increased density of Purkinje neurons and reduced density of granular layer in cerebellum. Similarly, the *deafwaddler* (*dfw*) and *wriggle mouse sagami* (*wri*) strains displayed similar phenotypes such as tremor and vestibular/motor imbalance. Both *dfw* and *wri* genes were reported to be associated with a mutation in PMCA2 gene [107,108,109]. Interestingly, despite prolonged accumulation of Ca^2+^ in the cytosol of Purkinje neurons, heterozygous PMCA2^+/−^ mice exhibited outwardly normal behavior but presented clear deficits in hindlimb coordination when challenged with exercise [110].

The first mutation in PMCA2 (V1143F) associated with congenital cerebellar ataxia has been recently identified by Vicario and coworkers [111]. In contrast to the hearing loss phenotype [105], the ataxia phenotype was generated without corresponding mutations in cadherin 23 and the hearing ability was fully retained. As V1143F substitution was located within CaM binding domain, it affected the interplay between mutated pump and CaM and the effect was particularly visible for full-length PMCA2 variant. Like other mutations, one of the consequences was prolonged duration of Ca^2+^ transients and compromised ability to maintain Ca^2+^ homeostasis in neurons. The list of PMCA mutations associated with cerebellar defects are summarized in Table 2.

**Table 2:** List of PMCA mutations and associated phenotype. Modified based on [112].

	Species	Mutation	Phenotype	Reference
PMCA2	Mouse	G283S (*Dfw*)	Vestibular/motor imbalance	[107]
	Mouse	I655N (*Elfin*)	Ataxia	[113]
	Mouse	S877F (*Obv*)	Ataxia	[114]
	Mouse	E629K (*Tmy*)	Ataxia	[115]
	Mouse	E412K (*Wri*)	Abnormal movements	[116]
	Human	V1143F	Ataxia	[111]
PMCA3	Human	G1107D	Ataxia	[93]
	Human	R482H	Ataxia	[97]
	Rat	R35C	Ataxia	[117]
	Human	G773R	Ataxia	[99]

There is substantial body of evidence to suggest that CaM-regulated PMCA isoforms play an important role in neuronal survival and synaptic transmission, thus contributing to several pathological states of the CNS. However, at the present stage of development, the exact molecular mechanisms by which the defective PMCA function leads to generation of disease phenotype is still under investigation. Plausibly, the answer lies in the regulation of Ca^2+^ within discrete plasma membrane microdomains hosting PMCA-associated signaling molecules with CaM, due to its universality as a Ca^2+^ decoding molecule, being central to the regulation of neuronal signaling.

### 3.2. PMCA-Interacting Proteins in Mental Diseases

Besides the well-known regulation of PMCA by CaM, the pump is also affected in less well-studied ways by multiple interacting partners that have been associated with many of the neurodegenerative diseases (Table 3) [118]. These interactions are thought to target PMCA to highly-specialized membrane microdomains, regulate pump activity or recruit it to multiprotein complexes—“signalosomes” responsible for orchestration of local Ca^2+^ signaling. The dynamic and fidelity of these interactions determined by structural differences further augments functional specialization of particular PMCA isoforms. For instance, it has been demonstrated that PMCA2b and PMCA4b interact with postsynaptic density protein-95 (PSD-95, not indicated in the table but widely discussed elsewhere [119,120,121,122]), which tether the pump to microdomains enriched in NMDA receptor [123]. Alterations in NMDA receptor are, in turn, frequently reported in schizophrenia, mood disorders, Huntington’s disease (HD), AD and substance-induced psychosis [124]. The functional coupling between NMDA receptor and PMCA would allow rapid response to local Ca^2+^ rises due to bring the pump to a local Ca^2+^ entry sites. The formation of tertiary PMCA/PSD95/NMDA receptor complexes could modulate the amplitude of Ca^2+^ increases and affect the neurotransmission. Such modulation has been recently showed for glutamate in a rat model of ketamine-induced psychosis [87]. As PMCA plays a pivotal role in the regulation of local Ca^2+^ fluxes, it cannot be ruled out that interaction with the binding partners may affect their clustering into large signaling complexes and influence the neurosecretory process.

## 4. The Sarco/Endoplasmic Reticulum Ca^2+^-ATPase (SERCA)

The SERCA pump is the product of a multigene family. It is a 110-kDa single polypeptide located in the sarco/endoplasmic reticulum (SR/ER) which primary role is to transport Ca^2+^ back to the internal stores. Mammals express three isoforms of the pump, called SERCA1 through SERCA3, and post-translational modifications increase the total number of identified subtypes to 10 [140]. The expression profile of individual variants is not only tissue-dependent, but also undergoes changes during development. The distribution of SERCA1 is limited to fast- and slow-twitch skeletal muscles, and the role of this pump is to accumulate calcium in SR of skeletal muscle. The alternative splicing of SERCA1 gene generates two variants which expression pattern is developmentally regulated. SERCA1b is predominantly expressed in neonatal stages, then is entirely replaced by SERCA1a in adults [141]. SERCA2 exists in two variants—SERCA2a and SERCA 2b [141]. SERCA2a is mostly found in cardiac and skeletal muscle with slow contractile characteristics, but also exhibits minor expression in the brain, where it is almost exclusively restricted to the Purkinje neurons of the cerebellum [142]. SERCA2a is considered to be involved in contraction and relaxation of cardiac muscle. SERCA2b is the most abundant variant expressed widely in all tissue types, including neurons. In the brain, SERCA2b expression has been identified in both, cerebrum, and cerebellum [143]. Moreover, SERCA2b is the only SERCA variant present in astrocytes [144]. Further studies confirmed that both variants of SERCA2 pump are present in substantia nigra—the structure involved in dopamine release [145]. In humans, there are six possible variants of SERCA3 [141]. However, due to the complex alternative splicing, their functional and structure aspects remain poorly understood. SERCA3a-3f are located in most tissues, especially in secretory cells. Because of predominant presence of SERCA3 in pancreatic β-cells, this isoform is recognized as being involved in metabolic homeostasis [146].

Despite high structural homology, all isoforms of SERCA possess different affinity for calcium and unique kinetic properties. For instance, SERCA2a has a two-fold lower affinity for [Ca^2+^] and shows two-fold higher velocity of Ca^2+^ transport compared to ubiquitous SERCA2b [147]. This translates into primary role of SERCA2a in cardiac function, whereas SERCA2b is considered more as a house-keeping form. In turn, among all SERCA isoforms the lowest affinity for calcium and the slowest turnover rate for Ca^2+^ uptake can be ascribed to SERCA3 [148]. This feature is a consequence of the distribution of this isoform in non-excitable cells. Accumulating evidence suggests that SERCA2 is ubiquitously present in different brain areas and therefore may be the isoform of paramount importance for neuronal function [143,149,150,151].

### 4.1. SERCA Pumps in Neuropathology

Disturbances in ER Ca^2+^ homeostasis can lead, among others, to ER stress and accumulation of unfolded or misfolded proteins, which are detected for most neurodegenerative diseases, including AD, PD, HD, and ALS [152,153,154]. It has been reported that truncated isoform of human SERCA1 (S1T) triggered and amplified ER stress response, leading to apoptosis [155]. Furthermore, the increase of human S1T protein expression has been demonstrated in sporadic AD-derived post-mortem brains and in a cellular AD model, confirming that S1T can induce neuroinflammatory response in vitro and in vivo [156].

An interesting observation was identification of several ATP2A2 mutations in autosomal dominant skin disorder—Darier’s disease (DD), a disorder frequently associated with several mental diseases (bipolar disorder, schizophrenia, affective psychosis, epilepsy) [157,158,159,160,161]. Numerous SERCA2 mutations in DD have been detected, including missense and nonsense types, which produced the insoluble truncated, misfolded and/or aggregated proteins, finally decreasing the amount of fully active SERCA. All these results indicate that ATP2A2 mutations may have pleiotropic effects on the brain as well as skin.

### 4.2. Calmodulin-Controlled Regulation of SERCA Pumps

Although the direct regulation by CaM has been shown only for PMCA, a growing body of evidence indicates that CaM can significantly, but indirectly, participate in modulation of SERCA activity. The well-recognized mechanism exists in cardiac and skeletal muscles and is based on regulation of SERCA activity by endogenous molecule—phospholamban (PLN) [162,163]. This small, 52-amino-acid transmembrane protein, which is expressed almost exclusively in muscle cells, in non-phosphorylated form binds SERCA2a, the muscles-specific variant, and lowers its affinity for Ca^2+^, thus attenuating transport rate by ~50% [164]. It was shown that PLN can inhibit SERCA2a and SERCA2b isoforms to the same extent [165]. Phosphorylation at Ser16 by PKA or by Ca^2+^-CaMKII at Thr17 causes PLN dissociation from SERCA, thereby removes its inhibitory effect, and increases Ca^2+^ uptake to the SR [166]. The decrease in the phosphorylation level of PLN by protein phosphatases PP1 and/or PP2B restores interaction between SERCA2a and PLN, inhibiting pump activity [167]. Although up to now the presence of PLN protein has not been confirmed in neurons, its expression was demonstrated in astrocytes what may have profound implications for the function of CNS.

Astrocytes, the important elements of glia, are an integral part of the CNS that couple the activity of neurons and blood-brain barrier (BBB). The proper function of BBB requires cooperation between endothelium, astrocytes, neurons, and extracellular matrix. An important role in a progression of neurological diseases is a disruption of the BBB integrity [168,169]. Degeneration of BBB has been confirmed in different disease—AD, PD, ALS, multiple sclerosis (MS), vascular dementia, stroke, hypoxia, or ischemia [170]. It leads to altered permeability with subsequent infiltration of serum components what can trigger abnormal intrinsic signaling pathways, including those involving calcium and/or CaM. Under physiological conditions astrocytes secrete neurotrophic factors, growth factors and cytokines that regulate neurogenesis, synaptogenesis, neuromodulation, and neuronal survival, but abnormal function of astrocytes has been observed in many neurodegenerative diseases [171,172,173]. For example, accumulation of α-synuclein detected in PD astrocytes clearly indicates their critical role in the course of disease [174]. It has been recently demonstrated that in α-synuclein-treated brain astrocytes, a leucine-rich repeat kinase 2 mutant G2019S (LRRK2-GS) can act through SERCA inactivation triggering ER stress [175]. LRRK2 is a multifunctional protein kinase, localized in the cytoplasm and associated with cellular membrane structures, that contains many domains capable of protein–protein interactions [176]. The GS mutation in the kinase domain of LRRK2 is one of the most common genetic basis of PD [177,178,179]. SERCA directly interacts with LRRK2-GS and, after translocation to the ER, forms persistently inactive SERCA–PLN complex. Disruption of SERCA function causes Ca^2+^ depletion from ER, finally leading to the cell death.

## 5. Secretory Pathway Ca^2+^-ATPase (SPCA)—The Golgi-Resident Ca^2+^/Mn^2+^ Pump

SPCA pump, as a member of the P2A subfamily, shares some common structural and mechanistic properties of SERCA [180], yet in addition to high Ca^2+^ affinity, SPCA also represents strong preference for Mn^2+^ ions. This Ca^2+^/Mn^2+^ transporter possesses unique structural elements in the N-terminus and transmembrane (TM) region determining orientation and selectivity of the ion transport during phosphoryl-transfer reactions. Particularly, SPCA pump is crucial for maintaining the sufficient supply of Mn^2+^ for glycosyltransferases and sulfotransferases in the Golgi lumen [181]. On the other hand, in the cytosol, SPCA pump prevents excessive accumulation of Ca^2+^ and Mn^2+^, while the overload of these ions may trigger neurotoxicity resulting in several neurological disorders [182].

In human, two SPCA isoforms, SPCA1 and SPCA2 are encoded by ATP2C1 and ATP2C2 genes, respectively. These isoforms share nearly 60% of sequence identity and they exhibit distinct expression pattern and tissue distributions in the human body [183]. Although SPCA1 is ubiquitously presented in the Golgi membranes of almost all mammalian tissues [184], it displays predominant localization in the brain where it helps to maintain appropriate physiological features of neurons including reception, conduction, and transmission of signals [185,186]. For SPCA2, its expression is more restricted to respiratory, gastrointestinal, genitourinary systems, as well as salivary and mammary glands [187,188]. The abundance and the specific role of SPCA2 in the brain is still a contentious issue, however its subcellular localization was confined mainly to the Golgi apparatus (GA) and the secretory vesicles [181,187]. Through alternative splicing at the 3′-end of human ATP2C1 pre-mRNA, four splice variants are generated, leading to additional SPCA1a-d isoforms with different length and sequence at the COOH-terminal domain. However, functional differences among these mature variants still remain largely unexplored [189].

SPCA pump is presumed to be composed of ten membrane-spanning helices (M1-M10) and cytosolic headpiece containing conservative motifs, which are critical for transport functions in a SERCA-like manner, including the Thr–Gly–Glu loop in A site, the phosphate-accepting Asp residue, the ATP- and FITC-binding region, and the Asp–Pro–Pro–Arg loop between the N and P sites. Unlike SERCA, SPCA pump lacks long cytoplasmic tail and elongated luminal loops linking some of the transmembrane domains, and importantly, only one calcium-binding region in SPCA protein overlaps with site II in the transmembrane domains of SERCA [183]. The research on the PMR1 yeast enzyme has shown that interface between Gln-783 and Val-335 in M6 and M4 domains can be critical for Mn^2+^ transport by SPCA pump [180]. Human SPCA1a/2 isoforms exhibit comparable Mn^2+^ affinity while SPCA1a displays significantly lower apparent Ca^2+^ affinity than SPCA2. Remarkably, SPCA1a displays a two-fold higher maximal ATPase activity in the presence of Ca^2+^ as compared to Mn^2+^ conditions, whereas SPCA2 shows similar maximal turnover rates for both ions. These differences in biochemical properties of SPCA isoforms seem to be determined, at least in part, by EF hand-like motif that is present in SPCA1a N-terminus, but absent in SPCA2. It is believed that such motif influences lower affinity and higher Ca^2+^ capacity of this pump promoting stronger Ca^2+^-dependent autophosphorylation. This feature may have physiological implications in cells with a high calcium load and/or fulfilling secretory functions [190].

### 5.1. SPCA Pumps in Neuropathology

The GA is associated with post-translational processing of proteins destined for secretion as well as their incorporation into the ER, lysosomes or plasma membranes. In the GA, calcium can reach ~300 μM, due to the action of SERCA and SPCA localized in this organelle [191,192]. Stored Ca^2+^ can be released mainly by IP_3_ receptors following extracellular stimuli. It is now widely accepted that GA, together with ER and mitochondria, plays an important role in the regulation of cytosolic Ca^2+^. It has been shown that the maintenance of the Ca^2+^ content provided by SPCA1 appears essential for the correct structure of the entire GA and for important functions of the secretory pathway.

Early study on SPCA1 indicated its vital role during brain development and closure of neural tube [185,193]. Additionally, downregulation of SPCA1 disrupted the proper processes of neuronal growth and differentiation, leading to altered GA morphology (like its fragmentation), as well as slowed down protein transport in the Golgi compartments [194]. In neural tissue SPCA1 exhibited an important role in the control of cytoskeletal dynamics in mice neuroepithelial cells and perturbation of calcium homeostasis impaired apical constriction during neural tube closure [195]. Since the GA is an important platform for a number of signaling cascades, inactivation of SPCA1 can also induce the disturbances in mitochondrial structure and metabolism, increasing their sensitivity to stress conditions [196]. Thereby, the abnormal function of the GA in several neuropathologies can be initiated by one or more aforementioned mechanisms. Moreover, GA in neurons is suggested to participate in modification of calcium signaling in some diseases, including AD, HD, or ALS [197].

A unique SPCA1 expression pattern has been described during ischemic/reperfusion brain injury (IRI), which was affected by pre-ischemic challenge. Whereas IRI induced the depression of SPCA-mediated calcium transport in hippocampus, ischemic preconditioning (IPC) had a partial protective effect on SPCA activity [198]. Similar relationship between the expression of SPCA1 and calcium concentration in neuronal cytoplasm and GA was observed during cerebral ischemia and reperfusion [199]. In naive ischemia, SPCA1 declined at early reperfusion, but increased in late reperfusion [200]. In rat brain cortex and hippocampus down-regulation of SPCA2 due to IRI has also been shown [201]. Based on these findings, it can be concluded that both SPCA isoforms may play a vital role in the GA stress during brain IRI, showing dual type of action. In preconditioned rat brain, SPCA may reduce calcium overload and the level of oxidative stress, but on the other hand, SPCA level was downregulated after prolonged ischemia.

Besides Ca^2+^ transport, SPCA also controls manganese homeostasis, which is important for brain development. Moreover, it represents the only known system for cellular Mn^2+^ detoxification. In rat brain, SPCA1 was upregulated following Mn^2+^ exposure, but impaired regulation of Mn^2+^ transport may trigger neuronal pathology. Although SPCA2 shows more restricted distribution, its relatively high level in brain could be more important taking into account the correlations between manganese neurotoxicity and PD [202,203]. High extracellular Mn^2+^ concentration exerted the toxic, inhibitory effect on SPCA activity in cultured mice neurons and glia [204]. In addition, since Mn^2+^ and Ca^2+^ can occupy the same ion-transport site, Mn^2+^ toxicity may also affect Ca^2+^ homeostasis. At elevated levels, Mn^2+^ can also compete with magnesium-binding sites in many functional proteins, enhance oxidative stress or lead to accumulation of intracellular toxic metabolites [205]. Finally, disturbances in ions homeostasis can induce ER and mitochondrial dysfunction, leading to neuronal and/or glial apoptosis. Although glial cells seem to be more resistant to Mn^2+^ poisoning than neurons, prolonged dyshomeostasis may result in fragmentation of the GA. The role of Mn^2+^ in the etiology of neurodegenerative diseases has been widely documented. Excess Mn^2+^ was shown to induce a neurological disorder with symptoms similar to PD [206]. Moreover, Mn^2+^ contributed to AD associated with impaired cognitive function and cognitive decline [207].

### 5.2. Calmodulin-Controlled Regulation of Calcium in Golgi Apparatus

Changes in the GA morphology observed in a number of neuronal diseases such as AD, ALS, and stroke include GA fragmentation and intracellular Ca^2+^ overload, which can lead to additional injury. These processes are also associated with specific brain areas, as well as with BBB dysfunction. Mechanisms underlying the neuropathological changes are observed in different cell types and frequently result in improper communication between brain structures, including cooperation between neurons and glial cells [208]. Astrocytes, which represent the prevalent glial cell types in mammalian brain, are responsible for maintenance of neurotransmitter and ion balance [209]. The active, bidirectional regulation of synapses by astrocytes has been shown to influence neuronal and synaptic functions accompanied by the altered Ca^2+^ circulation. Furthermore, astrocytes are an integral part of the BBB [210]. Interestingly, many transport systems responsible for balancing cytosolic Ca^2+^ concentration are under feedback control of Ca^2+^/CaM complex [211,212,213]. Up to now, the direct regulation of SPCA by CaM has not be detected, but there are several indirect CaM-induced processes that can affect the GA function. One of them is linked with production of nitric oxide (NO). The initial release of calcium forms a Ca^2+^/CaM complex, which binds to nitric oxide synthase (NOS), and actives the enzyme producing NO from L-arginine. The most important target for NO is guanylate cyclase (GC). High level of NO can contribute to excitotoxicity following a stroke and neurodegenerative diseases. In addition, NOS generates superoxide, which is involved in both cell injury and signaling [214].

It has been demonstrated that Ca^2+^ released after the GA fragmentation caused by pathological conditions triggers overactivation of eNOS in a Ca^2+^/CaM-dependent way. Moreover, increased Mn^2+^ levels in astrocytes elevated the expression of iNOS and activated soluble guanylate cyclase (sGC), which is suggested to be a causative factor for PD [215]. sGC expression and activity appear to be higher in the striatum than in any other brain regions [216]. The second interesting regulation by Ca^2+^/CaM comprises the activation of CaMKII, the enzyme controlling multiple signaling pathways in the brain. It should be noted that the effects of CaMKII may be reversed by antagonistic action of protein phosphatases, particularly PP2B, which is also a Ca^2+^/CaM-dependent enzyme. Phosphorylation of nNOS at Ser847 by CaMKII was shown to decrease NO generation, but increase superoxide generation [217]. Subsequently, reactive oxygen species and reactive nitrogen species may damage intracellular membranes of ER, GA and mitochondria what results in the leakage of Ca ^2+^ into the cytoplasm. Nitrosative/oxidative stress may also induce BBB rupture and impair metabolic network of astrocytes [218].

Ca^2+^ in the ER and the GA is mainly released through the inositol-1,4,5-trisphosphate receptor (IP_3_R) and ryanodine receptor (RYR) channels, which are also regulated by Ca^2+^/CaM complex [219,220]. The role of IP_3_Rs has been shown in ataxia and neurodegenerative diseases such as AD and HD [221]. Recent studies also suggest that alterations in the expression and function of RyRs are related to neurodegenerative diseases such as AD [222].

The functionality of GA appears to be more complex, especially in its ability to counterbalance excessive Ca^2+^ and/or Mn^2+^ concentration. Participation of several ion transporting systems and cooperation of intracellular organelles is required for adaptation to stress conditions that allows protection against neurotoxicity. The importance of cooperation between GA, ER, and mitochondria in maintenance of calcium homeostasis has been suggested in many studies. Under pathological conditions improper control of Ca^2+^ circulation, its excessive accumulation and disturbed mitochondrial energy production preclude the formation of physiological Ca^2+^ signaling networks leading to neurotoxicity and cell death.

## 6. Concluding Remarks

There is now substantial evidence that defective Ca^2+^ signaling is frequently observed in majority of mental and neurodegenerative diseases. Because one of the most distinguishable features of Ca^2+^ is ambivalence, the dysfunctions of neuronal Ca^2+^-ATPases have recently acquired growing awareness and prominence. So far, no massive and uncontrolled neuronal death due to pumps dysfunctions have been identified. Instead, they are usually linked to less severe phenotypic changes that possibly originate from mild alterations in Ca^2+^ concentration in subtle cellular compartments or domains. It should be underlined that at different steps of subsequent Ca^2+^-induced processes, the Ca^2+^/CaM complex plays a decisive control function. Up to now, the studies revealed only certain human diseases, mostly with genetic defects in Ca^2+^ pumps. These phenotypes have gained considerable attention, however an intensified research on animal models is soon expected to describe the global effects of nongenetic pump alterations as well. Undoubtedly, knowing the consequences of Ca^2+^ pumps dysfunctions will help to understand the importance of Ca^2+^ signaling in development of neuropathologies. In view of that, future work should reveal the relationship between PMCA, SERCA and SPCA and their integration into dynamic processes of neuronal death, adaptation, and repair. The special attention should be given to CaM-regulatory mechanisms as primarily responsible for neuronal signaling in physiological and pathological states. This may significantly contribute to the identification of therapeutic strategies centered on neuronal calcium pumps.

## Figures and Tables

**Table 1:** Properties of PMCA isoforms. Modified based on [1].

	PMCA1	PMCA2	PMCA3	PMCA4
**Tissue Distribution**	Ubiquitous	Restricted (brain)	Restricted (brain)	Ubiquitous
**Developmental Expression/Switch**	Isoform switch fetal/adult	Isoform switch fetal/adult	Isoform switch fetal/adult	Isoform switch fetal/adult
**Affinity CaM (K_d_ nM)**	40–50	2–4	8	3–40

**Table 3:** Protein interacting with PMCA. Modified based on [125]. AD—Alzheimer’s disease; BP—bipolar disorder; HD—Huntington’s disease; PD—Parkinson’s disease; SZ—schizophrenia, MDD—major depressive disorder, ALS—amytrophic lateral sclerosis, ASD—autism spectrum disorder.

A Protein Interacting with PMCA	Associated Disease	PMCA Domain Involved in the Interaction	A Protein Domain Involved in the Interaction	Functional Importance of the Interaction
NOS	AD, BP, MDD, ALS, anxiety, stroke, HD [126,127]	PDZ-domain binding sequence	PDZ domain	Decline in NOS activity, down-regulation of NO production
CASK	Microcephaly with pontine and cerebellar hypoplasia, X-linked intellectual disability, ASD [128]	PDZ-domain binding sequence	PDZ domain	Down-regulation the T- dependent transcriptional activity
CLP36	Williams-Beuren syndrome [129]	PDZ-domain binding sequence	PDZ domain	Pump translocation during platelet activation
MAGUK	AD, PD, stroke, X-linked mental retardation, BD, MDD, SZ [130,131,132]	PDZ-domain binding sequence	PDZ domain	Biding enables the localization of PMCAs in specific membrane domains and local control of Ca^2+^ concentration
Ania3/Homer	SZ, ASD, MDD, suicide attempt, cocaine dependence, opiate abuse [133]	PDZ-domain binding sequence	PDZ domain	Stabilization of PMCA in domains near the sites of calcium influx into the cell
Calcineurin	AD, HD, SZ, PD, ALS, BD epilepsy [134,135,136]	catalytic domain	Amino acids 58–143	Inhibition of the phosphatase activity of calcineurin, decrease in the activity of the transcription factor NFAT
Syntrophin α1	SZ, mild intellectual disability [137,138]	catalytic domain	Amino acids 399–447	The formation of a triple complex with PMCA and NOS-1 inhibits the production of NO
ε 14-3-3	AD, BP, PD, SZ [139]	the N-terminal region	Amino acids 2–92	Inhibition of PMCA activity
